# Finding a Benign Plasticizer to Enhance the Microbial Degradation of Polyhydroxybutyrate (PHB) Evaluated by PHB Degrader *Microbulbifer* sp. SOL66

**DOI:** 10.3390/polym14173625

**Published:** 2022-09-01

**Authors:** Jang Yeon Cho, Su Hyun Kim, Hee Ju Jung, Do Hyun Cho, Byung Chan Kim, Shashi Kant Bhatia, Jungoh Ahn, Jong-Min Jeon, Jeong-Jun Yoon, Jongbok Lee, Yung-Hun Yang

**Affiliations:** 1 Department of Biological Engineering, College of Engineering, Konkuk University, Seoul 05029, Korea; 2 Biotechnology Process Engineering Center, Korea Research Institute Bioscience Biotechnology (KRIBB), Cheongju 28116, Korea; 3 Green & Sustainable Materials R&D Department, Research Institute of Clean Manufacturing System, Korea Institute of Industrial Technology (KITECH), Cheonan 31056, Korea; 4 Department of Biological and Chemical Engineering, Hongik University, Sejong 30016, Korea

**Keywords:** polyhydroxybutyrate, plasticizer, biodegradation, *Microbulbifer* sp. SOL66

## Abstract

As a biodegradable plastic, polyhydroxybutyrate (PHB) has relatively poor mechanical properties, preventing its wider use. Various plasticizers have been studied to improve the mechanical properties of PHB; however, due to the slow degradation speed in the soil environment and lack of evaluation methods, studies on the degradation of PHB with plasticizers are rarely reported. In this study, by applying *Microbulbifer* sp. SOL66, which is able to degrade PHB very quickly, a benign plasticizer was evaluated with good properties and good degradability, not inhibiting microbial activities. Eight different plasticizers were applied with PHB and *Microbulbifer* sp. SOL66, PHB film containing 10% and 20% tributyl citrate showed significant biodegradability of PHB. It was confirmed that tributyl citrate could increase the speed of PHB degradation by *Microbulbifer* sp. SOL66 by 88% at 1 day, although the degree of degradation was similar after 3 days with and without tributyl citrate. By the analysis of microbial degradation, physical, chemical, and mechanical properties, tributyl citrate was shown not only to improve physical, chemical, and mechanical properties but also the speed of microbial degradation.

## 1. Introduction

In recent years, people have become more aware of environmental pollution and have made efforts to avoid the continuous use of non-degradable conventional plastics [1,2]. Bioplastics are a suitable alternative to disposable products and can be produced and degraded by microorganisms [3]. There are many kinds of bioplastics such as polylactic acid (PLA), poly(butylene adipate-*co*-terephthalate) (PBAT), and polyhydroxybutyrate (PHB) that can substitute non-degradable conventional plastics [4,5]. Since PLA and PBAT have superior properties, they are already being used little by little in various industrial fields such as packaging and agriculture industries [6,7].

In contrast to PLA and PBAT, which are not produced directly by microorganisms, PHB has the advantage that it can be produced directly through fermentation. Several microorganisms have been reported to have PHB accumulation capability, since PHB is a metabolite in the butanoate metabolism of microorganisms [8]. Moreover, PHB accumulators also express PHB depolymerase and can easily degrade PHB compared with PLA and PBAT. However, compared to PLA and PBAT, which are easily observed in daily life, PHB is not frequently observed. The main reason is that PHB is brittle: it does not stretch well and is easily torn, compared with other kinds of bioplastics [9,10,11,12].

There are many ways to strengthen PHB properties, but the most commonly used method is to use a plasticizer. A plasticizer is an additive added to change the physical properties of a material. When a plasticizer is added to a polymer such as plastic, the attractive force between the polymer chains is reduced, thereby increasing its flexibility [13]. Many kinds of plasticizers have already been added to non-degradable conventional plastics [14]. Since the addition of plasticizers greatly affects the physical properties of plastics, research on adding various plasticizers to bioplastics is being actively conducted [15,16]. Because most plasticizers are chemical substances with complex structures that are not related to microorganisms, there is a possibility that some plasticizers may adversely affect microorganisms [17]. In this case, the advantages of bioplastics may be weakened by the effect of plasticizers on microorganisms or their biodegradability. Therefore, carrying out research into finding a plasticizer that has good compatibility with bioplastic-degrading microorganisms is important. When carrying out such research, there are limitations of time and place when conducting all kinds of experiments in natural environments such as soil or ocean.

Therefore, in this study, plasticized PHB was prepared by adding various kinds of plasticizers to PHB, and their properties were compared through various analytical instruments such as differential scanning calorimetry (DSC), X-ray diffraction (XRD), thermogravimetric analysis (TGA), and universal testing machine (UTM). The effect of various plasticizers on the *Microbulbifer* sp. SOL66 (already been proven as a superior PHB degrading strain in our laboratory) was studied [18,19]. PHB degradation was also evaluated by studying the difference in the weight loss of PHB added with various kinds of plasticizers. This result suggests which plasticizer is suitable or unsuitable for use in PHB. By using *Microbulbifer* sp. SOL66 that can almost completely degrade PHB in 2–3 days, a benign plasticizer with good properties and good biodegradability could be selected very quickly, while overcoming the limitations of time and place.

## 2. Materials and Methods

### 2.1. Chemicals

All chemicals used in this study were of analytical grade. PHB pellets (Average MW: 550,000 g/mol) were obtained from Goodfellow Cambridge Ltd. (Huntingdon, UK). Chloroform and ethanol were obtained from Fisher Scientific (Hampton, NH, USA). Bis(2-ethylhexyl) adipate, bis(2-ethylhexyl) sebacate, lauric acid, triacetin, tributyl citrate, tributyl 2-acetylcitrate, l-Linalool, and geraniol were obtained from Sigma-Aldrich (St. Louis, MO, USA).

### 2.2. Toxicity Test of Various Plasticizers to the Growth of Microbulbifer sp. SOL66

To confirm the effect of various plasticizers on microbial cells, growth measurements were conducted under the effect of different concentrations of plasticizers. Bis(2-ethylhexyl) adipate, bis(2-ethylhexyl) sebacate, triacetin, tributyl citrate, tributyl 2-acetylcitrate, l-Linalool, and geraniol were used in concentrations from 0.25 mM to 16 mM. Only lauric acid was used in concentrations from 0.02 mM to 1.28 mM due to solubility. *Microbulbifer* sp. SOL66 cells were precultured in 5 mL marine broth (MB; Kisanbio, Seoul, Republic of Korea) in a shaking incubator at 37 °C, 200 rpm, for 24 h. Cells taken from the preculture were used to inoculate into the main culture process. In this process, pipetting robot (Integra, Le Locle, Switzerland) was used for the dilution of each plasticizer. Before the experiment, all kinds of plasticizers were dissolved in the HPLC grade ethanol to give a final concentration of 1.6 M of each plasticizer, except lauric acid. It cannot be dissolved significantly so a stock solution of 128 mM was prepared. All of the stock solutions were sterilized by using a filter (pore size, 0.2 μm). A 96-well cell culture plate (Thermo Fisher Scientific, MA, USA) was used for the culture process. MB liquid media 200 μL were inoculated with 2% of *Microbulbifer* sp. SOL66 in all the spotted wells, except for the first column. In the first column, 400 μL of MB liquid media containing 4% of *Microbulbifer* sp. SOL66 were spotted. After 4 μL of each plasticizer was added to the first column, a 96-well cell culture plate was set on the pipetting robot instrument. Then, according to the dilution sequence pre-arranged on this instrument, the solution was diluted two-fold while passing to the next column sequentially. In the dilution process, the mixing process through pipetting 5 times was also included for each dilution. After that, it was incubated in a shaking incubator at 37 °C, 200 rpm, for 24 h. The optical density after incubation was measured at 595 nm using a 96-well microplate reader (Tecan, Mannedorf, Switzerland) [20].

### 2.3. Degradation of PHB Containing Various Plasticizers under the Liquid Condition

For the liquid culture degradation test, PHB films with a thickness of 0.04 mm containing various plasticizers were prepared with 10% (*w*/*w*) and 20% (*w*/*w*) of concentrations. PHB pellets were placed in a glass bottle, into which 50 mL of chloroform was added. Then, each plasticizer was added to 10% and 20% of the total mass. The bottles were heated at 60 °C in a water bath until the contents were completely dissolved. Heated chloroform containing plasticized PHB was poured into a glass Petri dish in a fume hood, and a film was made by evaporating all of the solvents. Next, all films were cut by 20 mg each and then sterilized by immersing in 70% ethanol and drying in UV light. Then, each film was cultivated with the precultured *Microbulbifer* sp. SOL66 in 5 mL of MB liquid media [21]. After 2 days of cultivation at 37 °C, 200 rpm, optical density was measured with the same process described in the previous section. Degraded PHB films were recovered and washed with distilled water to remove impurities containing cell debris. In the case of time-dependent analysis, PHB films degraded by *Microbulbifer* sp. SOL66 after 1, 2, and 3 days of cultivation were recovered and treated with the same procedure. Recovered films were analyzed using a GC-MS instrument to measure weight loss.

### 2.4. GC-MS Analysis

The degree of degradation was calculated with GC-MS data. Before using the GC-MS instrument, fatty acid methyl ester (FAME) derivatization was conducted for preparing the GC-MS sample [21,22]. Then, 1 mL of methanol/sulfuric acid (85:15 *v*/*v*) and 1 mL of chloroform were mixed in each glass vial containing lyophilized PHB films. Additionally, each vial was sealed with a screw cap and Teflon tape and was heated using a heating block at 100 °C for 2 h for FAME derivatization. After the heating process, the samples were subsequently cooled at a low temperature for approximately 10 min, 1 mL of HPLC-grade water was added to each vial for making layer separation, and the samples were gently vortexed for 1 min. The bottom layer (chloroform) was transferred to a new 1.5 mL e-tube containing sodium sulfate anhydrous to remove water. Each sample was filtered (pore size, 0.2 μm) into a clean borosilicate glass tube. The prepared sample was analyzed by GC-MS (Perkin Elmer, Waltham, MA, USA) equipped with a fused silica capillary column (Elite-5 ms, 30 m × 0.25 mm i.d. × 0.25 µm) and subjected to a linear temperature gradient for analysis (40 °C for 1 min, increased at 15 °C/min to 120 °C and held for 2 min, and then increased at 10 °C/min to 280 °C and held for 10 min). The injector port temperature was 250 °C. Mass spectra were obtained by electron impact ionization at 70 eV, and scan spectra were obtained within the range of 45–450 m/z. Selected ion monitoring was used for the detection and fragmentation analysis of the major products. A standard curve was constructed using each kind of film to quantify the degraded PHB films. When a plasticizer was added to each film, the degree of degradation was calculated by considering the ratio.

### 2.5. Chemical and Mechanical Analysis

DSC analysis of the plasticized PHB films and non-plasticized PHB films was performed using the Discovery DSC instrument (TA Instruments, New Castle, DE, USA) at temperatures ranging from −80 °C to 200 °C, while the heating and cooling rate was 10 °C/min in an N_2_ atmosphere [23,24,25].

To analyze the crystalline/amorphous characteristics of plasticized and non-plasticized PHB films, XRD analysis was performed using an X-ray diffractometer (Rigaku Corporation, Tokyo, Japan) with a Cu Kα (λ = 1.54 Å) source, operating at 40 kV and 30 mA as the applied voltage and current, respectively. The 2θ range was from 3° to 60° at a scanning rate of 2°/min [25,26,27,28].

Thermogravimetric analysis (TGA) was performed using a TGA N-1000 (Scinco, Seoul, Korea). Samples were heated from 30 °C to 700 °C, at a heating rate of 10 °C/min under nitrogen flow (20 mL/min) [29,30,31].

Mechanical properties of plasticized and non-plasticized PHB films were measured using an EZ-SX universal testing machine (Shimadzu, Kyoto, Japan) [32,33,34]. Before measurement, PHB films were cut into a rectangular sample of size 60 mm × 10 mm (length × width). Thickness, which depends on the presence and amount of plasticizer, was measured using an 1111–100 A mini digital caliper (Insize, Loganville, GA, USA). Data were collected at a strain rate of 20 mm/min. After measurement, tensile strength, young’s modulus, and elongation at break were calculated from a plot formed by TRAPEZIUM X software.

### 2.6. SEM Analysis

Scanning electron microscopy (SEM) analysis was performed to analyze the surface changes in the plasticized and non-plasticized PHB films after degradation by *Microbulbifer* sp. SOL66. Recovered PHB films after 2 days of degradation by *Microbulbifer* sp. SOL66 were washed and lyophilized before SEM analysis. The lyophilized films were then coated with gold dust at 5 mA for 300 s, and back-scatter electron images were obtained using a TM4000Plus SEM instrument (Hitachi, Tokyo, Japan) at a voltage of 5 kV [35].

### 2.7. GPC Analysis

Gel permeation chromatography (GPC) analysis was performed to detect the molecular weight changes in plasticized and non-plasticized PHB films after degradation by *Microbulbifer* sp. SOL66. The sample preparation process was carried out with the same method as our previous research [21]. A high-performance liquid chromatography (HPLC) apparatus was used for measuring molecular weight change, consisting of a loop injector (Rheodyne 7725i), an isocratic pump with dual heads (YL9112), column oven (YL9131), columns (Shodex, K-805, 8.0 mm I.D. × 300 mm; Shodex, K-804, 8.0 mm I.D. × 300 mm), and RI detector (YL9170). Sixty microliters of the sample from which bubbles were removed were injected. Only twenty microliters of injected samples were used as analytes for analysis in the injector part. The mobile phase was chloroform, and this flow rate was maintained at 1.0 mL/min. The temperature was maintained at 40 °C for analysis. The data were analyzed using YL-Clarity software for a single YL HPLC instrument (YOUNG IN Chromass, Anyang, Republic of Korea). The molecular weight was calculated with polystyrene standards ranging from 5000 to 2,000,000 g/mol [35].

## 3. Results

### 3.1. Comparison of Properties of Various Plasticizers

Eight kinds of plasticizers containing bis(2-ethylhexyl) adipate, bis(2-ethylhexyl) sebacate, lauric acid, triacetin, tributyl citrate, tributyl 2-acetylcitrate, L-Linalool, and geraniol which were often used for increasing properties of conventional plastics and bioplastics were included in the PHB film. As listed in (Table 1), all of the plasticizers that have been already reported for plasticizing PHB have very complex molecular structures and various molecular weights. Bis(2-ethylhexyl) adipate is the most commonly used plasticizer [36] because it can improve the flexibility of plastics and it has been regarded as a safe plasticizer. However, there is a precedent in which hepatic, brain, and cardiac injuries were found in rats when exposed to a large amount of this substance [37]. Although bis(2-ethylhexyl) sebacate is relatively less dangerous than bis(2-ethylhexyl) adipate, it is still not a recommended plasticizer because it is still dangerous [38,39]. Additionally, lauric acid can be used as a plasticizer because it forms a hydrogen bond when incorporated into the plastic, decreasing the force of each plastic molecule, resulting in a decrease in glass transition temperature [40,41]. Triacetin is a well-known plasticizer for PHB due to its improved biodegradability of PHB [42,43]. Tributyl citrate and tributyl 2-acetylcitrate are widely used as plasticizers in bioplastics such as PHB due to their high biocompatibility [27,44,45]. L-Linalool and geraniol are natural monoterpenoids, which are components of essential oils derived from plants, and have a unique scent. Since it can be obtained from nature rather than chemically synthesized, it has the advantage of being environmentally friendly [46]. Since all plasticizers have their strengths and weaknesses, both physical properties and biodegradability were considered to select the most appropriate plasticizer for PHB.

### 3.2. Effect of Various Plasticizers on Microbulbifer sp. SOL66 Cell

**Figure 1:**
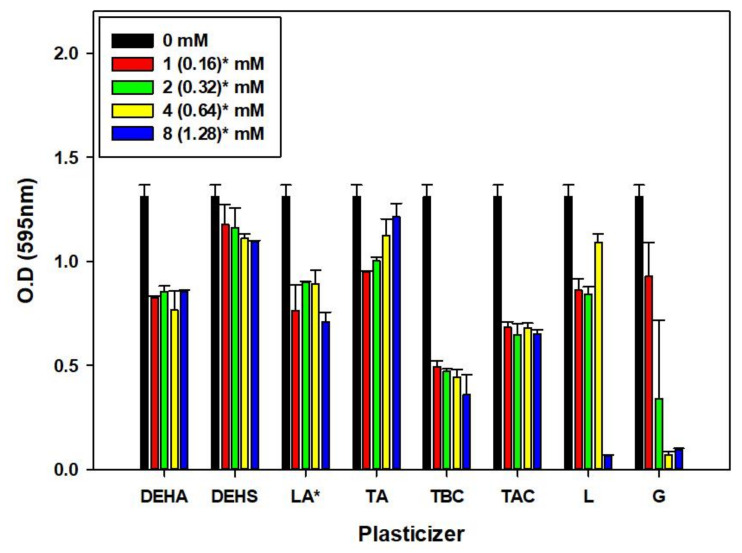
Growth tendency with the addition of various plasticizers. The cell growth pattern of *Microbulbifer* sp. SOL66 was expressed as optical density with the addition of plasticizers at concentrations of 1 mM, 2 mM, 4 mM, and 8 mM, lauric acid* was added at 0.16 mM, 0.32 mM, 0.64 mM, and 1.28 mM due to solubility issues.

Before comparing biodegradability, a toxicity test on bacteria *Microbulbifer* sp. SOL66 was conducted with all 8 plasticizers. If a plasticizer that prevents cell growth is used, the biodegradation process using microorganisms is disrupted. So, compatibility between the plasticizer and *Microbulbifer* sp. SOL66 was confirmed. Although the toxicity of a plasticizer does not impede all biodegradation processes, confirmation of toxicity can provide a certain reference point for evaluating the suitability of the plasticizer for PHB, which can be easily degraded by *Microbulbifer* sp. SOL66. As a result of the toxicity test with various plasticizers, the effect of each plasticizer addition on the growth of *Microbulbifer* sp. SOL66 was varied (Figure 1).

In all cases, the growth of *Microbulbifer* sp. SOL66 was reduced when a plasticizer was added. However, in the case of triacetin, the cell growth gradually increased as the concentration increased. Except for this, the addition of bis(2-ethylhexyl) adipate, bis(2-ethylhexyl) sebacate, lauric acid, tributyl citrate, and tributyl 2-acetylcitrate showed a generally decreasing tendency as the concentration increased. When L-Linalool and geraniol, which are natural monoterpenoid plasticizers, were added, *Microbulbifer* sp. SOL66 did not grow at all when more than 8 mM and 4 mM were added, respectively. However, this does not mean that plasticizers that reduce or inhibit microbial growth are not good when added to PHB. It suggests that plasticizers may adversely affect cell growth when applied directly to cells. The impact of toxicity as a molecule might be different from the role inside of the polymer.

### 3.3. Thermal Properties Analyzed by DSC

To confirm the change in chemical properties by the addition of plasticizer, DSC analysis was performed to measure the glass transition temperature (*T_g_*), melting temperature (*T_m_*), and crystallization temperature (*T_c_*). In general, it is known that adding a plasticizer creates free space between the polymers, thereby reducing the *T_g_* value and softening the physical properties by allowing the components of the polymer to move freely [13,47,48]. The DSC result showed that the *T_g_* value of plasticized PHB film is low compared with non-plasticized PHB, except for PHB film containing 20% of L-Linalool (Table 2). It can be supposed that the addition of various kinds of plasticizers increased the softness of PHB. Not only the *T_g_* value but also the *T_m_* value and *T_c_* value changed according to the addition of the various plasticizers, but the crystallinity value (*X_c_*) derived from the enthalpy change at melting temperature (Δ*H_m_*) is more important in analyzing the properties. *X_c_* value was calculated using Equation (1) [23,29,30].

(1) $$X_{c}\left(\%\right)=\frac{\Delta H_{m}}{\Delta H_{m}100\%}\times \frac{100}{W_{PHB}}\left(\%\right)$$

The melting enthalpy of a 100% crystalline 3-hydroxybutyric acid (Δ*H_m_* 100%) was reported to be 146 J/g and *W_PHB_* means the weight fraction of PHB in the plasticized PHB [49]. By using this, *X_c_* values of plasticized PHB were calculated. The crystallinity of non-plasticized PHB was 43.1%, whereas the crystallinity of plasticized PHB was increased, except when 20% of bis(2-ethylhexyl) sebacate and 10% of L-Linalool were added to PHB. This is because adding a plasticizer improves the mobility of the polymer chain and promotes crystallization [44].

### 3.4. Effect of Various Plasticizers on the Biodegradability of PHB by Microbulbifer sp. SOL66

**Figure 2:**
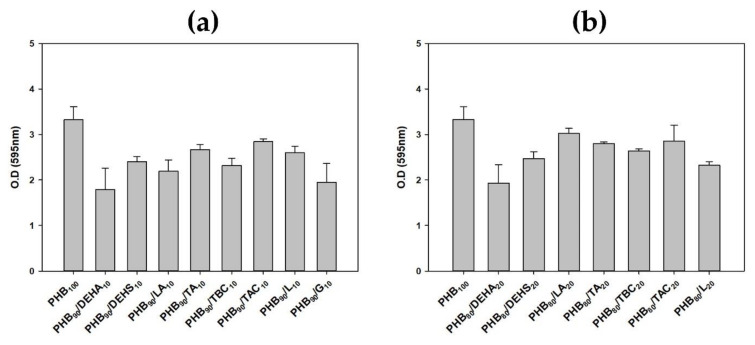
The growth pattern of *Microbulbifer* sp. SOL66 cultured with (**a**) 10% plasticized and (**b**) 20% plasticized PHB films.

After confirming the toxicity of various plasticizers as molecules themselves, the effect of plasticizers inside of PHB on the growth of cells was compared. In addition, the biodegradability of plasticized PHB was compared to screen the optimal plasticizer for better quality PHB that can be degraded by *Microbulbifer* sp. SOL66. Eight kinds of plasticizers were added to PHB film with 10% and 20% concentrations, respectively. After the sterilization process, plasticized PHB films were cultured together with *Microbulbifer* sp. SOL66 in a 37 °C shaking incubator for two days. After cultivation, the growth expressed as optical density was first measured to confirm the effect of the plasticizer contained in the PHB film on the *Microbulbifer* sp. SOL66 cells. In the case of culturing the PHB films containing 10% of the plasticizers, growth itself was reduced compared to the case of culturing the PHB film without the plasticizer as a whole (Figure 2a). However, there was no case where growth did not occur. On the other hand, even when the PHB films containing 20% of the plasticizers were cultured, the growth pattern was similar depending on the type of added plasticizer, but in the case of culture of the PHB film containing 20% of geraniol, *Microbulbifer* sp. SOL66 did not grow (Figure 2b). The result of completely inhibiting the growth of *Microbulbifer* sp. SOL66 when 20% of geraniol was included in the film was similar to the previous toxicity test, suggesting geraniol is not a suitable plasticizer for PHB when the evaluation of PHB degradation is conducted with *Microbulbifer* sp. SOL66., but the growth pattern when the plasticizer was included in the film was different from that when the plasticizer was directly applied to microbial cells.

**Figure 3:**
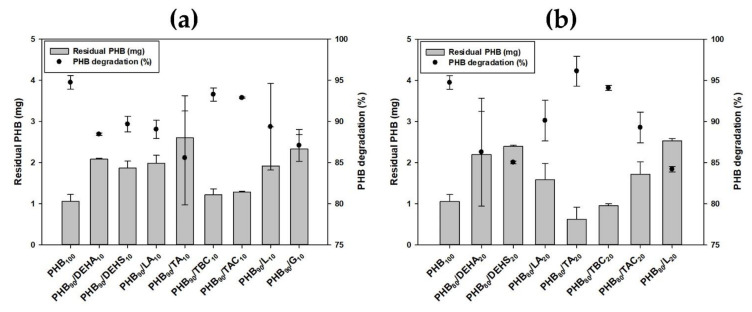
The comparison of degradation of (**a**) 10% plasticized and (**b**) 20% plasticized PHB films by *Microbulbifer* sp. SOL66.

As a result of analyzing the degree of degradation of the PHB film containing various plasticizers through GC-MS, when most plasticizers were added, the degree of degradation tended to decrease. In the case of degradation of PHB film containing 10% of plasticizers, the biodegradability of tributyl citrate and tributyl 2-acetylcitrate was similar to that of the film without plasticizers (Figure 3a). When 20% of plasticizers were included, the biodegradability of triacetin and tributyl citrate was comparable to that of the non-plasticized PHB (Figure 3b). When 20% of triacetin was added, the degree of degradation was higher than that of the non-plasticized PHB, but in the case of 10%, the degree of degradation was not very high. On the other hand, in the case of tributyl citrate, since it showed excellent robustness of biodegradability in both 10% and 20% plasticized PHB, it could be concluded that tributyl citrate was suitable as a plasticizer to be added to PHB, which is greatly degraded with *Microbulbifer* sp. SOL66, suggesting the toxic effect of plasticizer itself and degradation of plasticized PHB is different.

### 3.5. Comparison of Other Properties with Using Various Analytical Instruments

**Figure 4:**
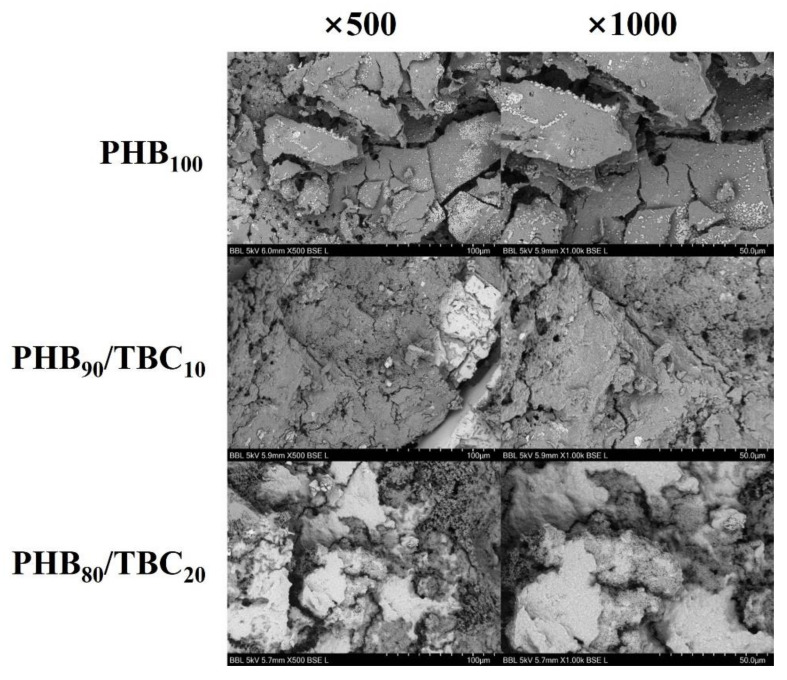
Representative images of surface changes observed by scanning electron microscopy. Comparison of differences in surface changes after two days of degradation by *Microbulbifer* sp. SOL66 through observation of 500 and 1000 magnifications.

As PHB with tributyl citrate showed higher degradation than PHB without tributyl citrate, the surface change with the addition of 10% and 20% of tributyl citrate after two days of degradation by *Microbulbifer* sp. SOL66 was compared using SEM. Since the surfaces of the non-plasticized PHB film and the plasticized PHB film before degradation were similar, the surface area characteristics of the film could be confirmed by checking the surface after degradation. The SEM result showed that the non-plasticized PHB film looked as if degradation had progressed as the cracked cross-section was split at an angle, and the cross-sectional portion where the tributyl citrate-added film was degraded was curved rather than angled (Figure 4). Through the comparison of the parts where the degradation has progressed in this way, it was possible to visually compare the difference in the physical properties of the surface.

Next, the change in molecular weight according to the addition of tributyl citrate before and after degradation by *Microbulbifer* sp. SOL66 was compared. As indicated in (Table 3), there was little difference between the number average molecular weight (*M_n_*) values of plasticized and non-plasticized PHB films before degradation by *Microbulbifer* sp. SOL66. However, there was a slight difference after 2 days of degradation. When tributyl citrate was added, the number average molecular weight decreased more than when it was not added. Conversely, polydispersity index (PDI) values after degradation were higher when tributyl citrate was added. Through the difference in molecular weight change, it was found that adding tributyl citrate to the PHB film could cause a change in degradation by *Microbulbifer* sp. SOL66.

**Figure 5:**
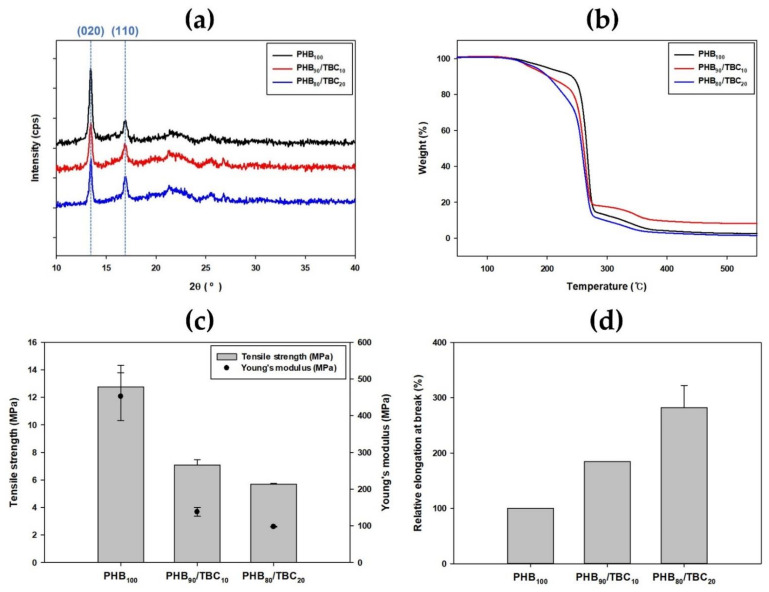
Changes in various properties according to the addition of tributyl citrate. Comparison of (**a**) XRD spectra and (**b**) thermo gravimetric analysis (TGA) result. Mechanical properties change expressed as (**c**) tensile strength (MPa), Young’s modulus (MPa), and (**d**) relative elongation at break (%) measured using a universal testing machine (UTM).

To find out the reason for high microbial degradation under the influence of plasticizers, XRD spectra of non-plasticized PHB film and PHB film plasticized with tributyl citrate were presented and compared (Figure 5a). The shape of the spectra was similar with or without plasticizer addition. In all three spectra, two strong peaks were commonly observed at 2θ = 13.5 and 2θ = 16.9, which are assigned with (020) and (110) diffraction planes. These peaks correspond to orthorhombic α-form crystals of PHB [50]. However, when a plasticizer was added, the intensities of these two peaks were slightly changed, meaning that crystal structures were slightly changed.

Next, the pattern of thermal degradation according to the presence or absence of the plasticizer was compared through the TGA results (Figure 5b). The pattern of abruptly decreasing weight near 250 °C was similar, but when tributyl citrate as a plasticizer was added, the weight was already greatly reduced before entering the rapidly decreasing section [29]. It was confirmed that the stability to heat decreased according to the concentration of the plasticizer added. Additionally, the higher the concentration of tributyl citrate, the lower the stability was. This is because plasticizer loss occurs when a high temperature is applied [27].

Lastly, the change in mechanical properties expressed as tensile strength, Young’s modulus, and relative elongation at break were compared through the universal testing machine results. When tributyl citrate as a plasticizer was added, tensile strength and Young’s modulus were decreased by more than half (Figure 5c). However, the decrease in both values according to the increase in the concentration of the plasticizer was not very large. On the other hand, there was a large difference in the length of the film stretched (Figure 5d). When the elongation of the film without the addition of tributyl citrate was set to 100, and a relative comparison was conducted, the elongation was nearly doubled when 10% of tributyl citrate was included in PHB film, and it was almost tripled when 20% of tributyl citrate was included. This is because the addition of tributyl citrate as a plasticizer reduced the intermolecular interactions of PHB [51].

### 3.6. Time-Dependent Analysis of PHB Degradation

**Figure 6:**
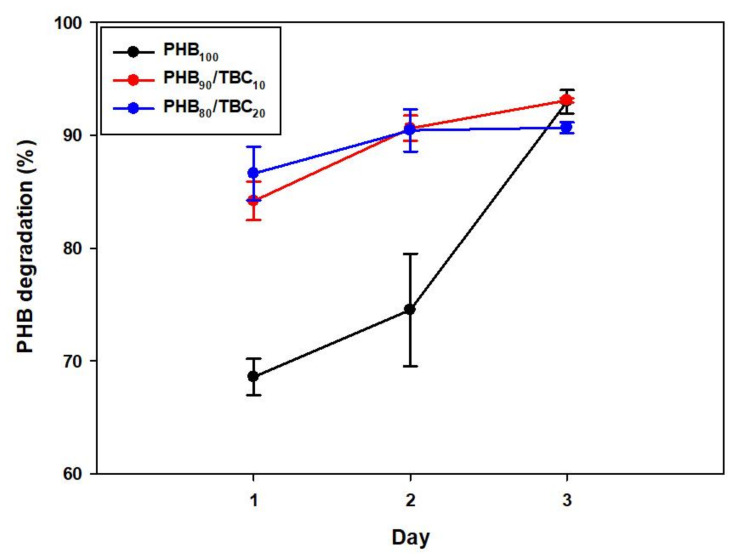
Time-dependent analysis of PHB biodegradation by *Microbulbifer* sp. SOL66. Comparison of the degradation rate of non-plasticized PHB film, and tributyl citrate plasticized PHB films.

Finally, the time-dependent degradation rate of non-plasticized PHB film, PHB film including 10% tributyl citrate, and PHB film including 20% tributyl citrate was compared (Figure 6). These films are degraded by *Microbulbifer* sp. SOL66 for 1, 2, and 3 days were recovered and analyzed. After 3 days, the final degree of degradation was similar to more than 90%. However, non-plasticized PHB film was degraded by about 70% after 1 day of degradation and was almost completely degraded as the degree of degradation increased gradually, whereas the plasticized PHB films showed a very high degree of degradation of more than 80% even after one day of degradation. It suggests that tributyl citrate not only changes physical properties and increases the elongation properties of the PHB film, but is also very excellent in terms of biodegradability by *Microbulbifer* sp. SOL66.

## 4. Conclusions

Interest in biodegradable bioplastics has increased significantly as a way to reduce environmental pollution caused by non-degradable conventional plastics. However, unlike PBAT, which stretches well, PHB is brittle and is easily broken when a force is applied. In the case of PHB, its main advantage is its eco-friendly production process, as it can be produced and degraded by microorganisms. To overcome its property-related shortcomings, various types of plasticizers can be added to PHB.

Our study concluded that in the presence of plasticizer tributyl citrate, the degree of degradation was excellent compared to other plasticizers, and the robustness of the degradation rate was excellent even when the concentration of the plasticizer was increased. In addition, this suggests that there is a speedy screening process of plasticizers for PHB using *Microbulbifer* sp. SOL66, which has superior PHB degradation ability. Tributyl citrate plasticizer can be used to prepare bioplastic to have better qualities with a higher biodegradation rate, which can help to manage emerging plastic-related issues.

## Figures and Tables

**Table 1:** Molecular properties and applied references of plasticizers used in this study.

Plasticizer	Molecular structure	Molecular formula	Molecular Weight (g/mol)	References
Bis(2-ethylhexyl) adipate (DEHA)	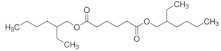	C_22_H_42_O_4_	370.57	[36,37]
Bis(2-ethylhexyl) sebacate (DEHS)	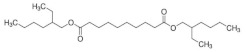	C_26_H_50_O_4_	426.67	[38,39]
Lauric acid (LA)	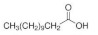	C_12_H_24_O_2_	200.32	[40,41]
Triacetin (TA)	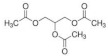	C_9_H_14_O_6_	218.20	[42,43]
Tributyl citrate (TBC)	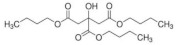	C_18_H_32_O_7_	360.44	[44,45]
Tributyl 2-acetylcitrate (TAC)	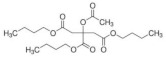	C_20_H_34_O_8_	402.48	[27,45]
L-Linalool (L)	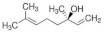	C_10_H_18_O	154.25	[46]
Geraniol (G)	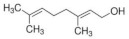	C_10_H_18_O	154.25	[46]

**Table 2:** Thermal properties of plasticized PHB analyzed by using DSC.

Plasticizer	Concentration (%)	*T_g_* (°C)	*T_m_* (°C)	*T_c_* (°C)	Δ*H_m_* (J/g)	*X_c_* (%)
Control	-	51.1	166.7	112.8	63.0	43.1
Bis(2-ethylhexyl) adipate (DEHA)	10	48.2	167.0	112.2	64.9	49.4
	20	46.8	163.0	110.5	56.1	48.0
Bis(2-ethylhexyl) sebacate (DEHS)	10	47.7	167.5	112.6	62.2	47.3
	20	48.5	166.2	112.1	50.3	43.1
Lauric acid (LA)	10	40.6	166.3	109.6	70.9	53.9
	20	40.2	160.5	104.0	61.3	52.5
Triacetin (TA)	10	44.0	165.0	110.9	67.7	51.5
	20	42.0	159.1	107.8	62.4	53.4
Tributyl citrate (TBC)	10	46.4	166.8	111.2	66.9	50.9
	20	42.9	164.0	108.5	51.8	44.3
Tributyl 2-acetylcitrate (TAC)	10	49.1	168.6	112.0	68.5	52.1
	20	45.1	161.0	105.9	76.0	65.0
L-Linalool (L)	10	49.7	169.0	113.5	50.5	38.4
	20	52.6	171.4	116.3	55.9	47.8
Geraniol (G)	10	44.3	166.2	112.1	61.3	46.7
	20	40.7	155.1	104.5	60.4	51.7

**Table 3:** Comparison of molecular weight change before and after degradation by *Microbulbifer* sp. SOL66 according to the addition of 0%, 10%, and 20% of tributyl citrate.

Day	TBC Concentration (%)	*M_n_* × 10^5^	PDI
0	0	5.33	1.46
	10	5.39	1.44
	20	5.33	1.44
2	0	0.29	1.56
	10	0.27	1.74
	20	0.26	1.67

## Data Availability

Not applicable.
